# Frequency-tunable continuous-wave random lasers at terahertz frequencies

**DOI:** 10.1038/s41377-019-0152-z

**Published:** 2019-05-01

**Authors:** Simone Biasco, Harvey E. Beere, David A. Ritchie, Lianhe Li, A. Giles Davies, Edmund H. Linfield, Miriam S. Vitiello

**Affiliations:** 1 NEST, CNR – Istituto Nanoscienze and Scuola Normale Superiore, Piazza San Silvestro 12, 56127 Pisa, Italy; 2 Cavendish Laboratory, University of Cambridge, Cambridge, CB3 0HE UK; 3 School of Electronic and Electrical Engineering, University of Leeds, Leeds, LS2 9JT UK

**Keywords:** Quantum cascade lasers, Electronics, photonics and device physics

## Abstract

Random lasers are a class of devices in which feedback arises from multiple elastic scattering in a highly disordered structure, providing an almost ideal light source for artefact-free imaging due to achievable low spatial coherence. However, for many applications ranging from sensing and spectroscopy to speckle-free imaging, it is essential to have high-radiance sources operating in continuous-wave (CW). In this paper, we demonstrate CW operation of a random laser using an electrically pumped quantum-cascade laser gain medium in which a bi-dimensional (2D) random distribution of air holes is patterned into the top metal waveguide. We obtain a highly collimated vertical emission at ~3 THz, with a 430 GHz bandwidth, device operation up to 110 K, peak (pulsed) power of 21 mW, and CW emission of 1.7 mW. Furthermore, we show that an external cavity formed with a movable mirror can be used to tune a random laser, obtaining continuous frequency tuning over 11 GHz.

## Medical lasers: Fine-tuned continuous wave random lasers in the terahertz region

A compact, tunable laser that operates at terahertz-frequencies could prove valuable in medical and diagnostic applications such as monitoring blood flow during surgery or for imaging reconstructions. Unlike X-rays, terahertz radiation does not damage living tissues, and therefore shows promise for high-resolution spectroscopy. Miriam Vitiello at the CNR-Nanoscience Institute, Italy, and co-workers designed a compact, terahertz-frequency random laser capable of pulsed and continuous-wave operation. Random lasers do not require specially-designed architectures like conventional lasers to amplify light; instead they use scattering from a highly-disordered gain medium alone, resulting in low spatial coherence and, potentially, improved quality imaging. Vitiello’s team incorporated a double-metal resonator randomly patterned with air holes in their design, allowing control over interference patterns and pulsing. Placing a movable mirror above the resonator’s top layer allowed fine spectral tuning of the laser output.

## Introduction

The terahertz (THz) region of the electromagnetic spectrum, loosely defined to span the frequency range between 300 GHz and 10 THz, bridges the domains of optics and electronics. It has applications across the fields of physics, chemistry, biology, and medicine—from metrology, spectroscopy, biomedical imaging, chemical detection, environmental monitoring, and security through information and communication technologies^1^.

Quantum cascade lasers (QCLs) emitting at THz frequencies^2^ have undergone rapid evolution since their first demonstration^3^ and in the last few years have become the best performing compact THz frequency source in terms of their output power and differential efficiency^4–7^, broad emission spectrum^8^, capability for beam shaping^9^, frequency tunability^10^, and low power consumption. Furthermore, photonic engineering, combined with new resonator concepts, have enabled the performance of THz QCLs to be designed with a high level of control, offering a flexible platform to tailor the emission spectrum, beam profile, and output power. Highlights have included the demonstration of one-dimensional laser cavities as third-order distributed feedback (DFB) lasers^11^, bi-periodic DFB lasers^12^, sinusoidal wire lasers^13^, graded photonic heterostructure lasers^14^, two-dimensional (2D) resonators for use in concentric-circular-grating DFB lasers^15^, and photonic crystal lasers^16–18^. Quasi-crystal lasers^19,20^ have additionally recently been used to achieve a remarkably high power extraction and good beam collimation, both in single-mode^19^ and multimode^20^ regimes.

It is further possible to engineer photonic properties using disordered systems. Such ‘random’ optical systems are amongst the most complex structures in photonics, displaying fascinating properties owing to their inherent structural disorder. Random light propagation has been widely investigated theoretically and experimentally^21,22^ in systems that include semiconductor powders^23^, optical fibres^24^, novel photonic glass materials^25^, and biological tissues^26^. In all cases, disorder induces intense multiple elastic scattering of light wavelets, leading to the formation of either extended or localized states of light inside the material known as Anderson states^27^. Tunable random laser emission in the visible spectrum has been demonstrated by varying the resonator geometry, boundary and absorbing conditions and scattering size/distribution, by tuning their spatial and temporal coherence^28^ or, post-processing, magnetically^29^, thermally^30^, through stretching^31^, or by electric-field modulation^32^.

Random lasers (RLs) strongly differ from conventional lasers, which traditionally comprise a gain medium enclosed in an optical cavity to produce feedback. Although RLs also require an active medium, this no longer needs to be embedded in a specially designed cavity since the feedback mechanism arises from multiple elastic scatterings in the highly disordered structure^33^. In RLs, the emitted photons can be amplified and scattered many times in the gain medium, preserving the phase relation of the wavelets. This results in a rich interference scheme that determines both the frequency and the spatial distribution of the electromagnetic radiation, with each optical mode having a different degree of localization. This complex interplay between the intrinsic disorder and the nonlinearity of a random active medium,^34–36^ additionally gives rise to many interesting physical phenomena, such as gain competition and nonlinear wave -mixing of the optical modes^37^.

Random lasing has been demonstrated in an optically pumped suspended micro-particle laser dye^38^, fine powders^39^, bone tissues^40^ and, more recently, in electrically pumped QCLs, operating in the mid-IR^41^ and THz frequency regions^42–44^ of the electromagnetic spectrum. For the latter, different device geometries exploiting air pillars^42^, semiconductor pillars^43^, or a combination of semiconductor and metal pillars^44^ have been used, but in all cases, patterning was achieved by etching through the entire active region. The resulting RLs emitted over a 300–400 GHz bandwidth, with peak optical powers ranging from hundreds of microwatts^44^ to few tens of milliwatts^42^ and with radiative outcoupling efficiencies *η*_r_ < 10%. However, operation has been observed only at low duty cycles (up to 4%) and at low (5 K) operating temperatures because of poor thermal management. Large device areas are indeed needed in these architectures, to ensure that there is sufficient gain in the radiative optical modes following fabrication of the deep-etched pillars.

Miniaturized, electrically pumped, continuously tunable RLs operating in a continuous wave (CW) regime are necessary for many spectroscopic and multicolour imaging applications across the THz frequency range. However, CW operation of an RL has yet to be demonstrated at THz frequencies, whether optically or electrically pumped, or indeed in the infrared part of the electromagnetic spectrum.

In this work, we have exploited a new resonator geometry in which a 2D random distribution of air holes, patterned into the top metal layer of a double-metal resonator, is combined with irregular borders to confine the active region. Patterning is, however, implemented only in the upper metal and in the highly doped semiconductor cladding, leaving the active region core unperturbed. We thus overcome the previous technological limitations of THz RLs, enabling us to demonstrate the first multimode CW emission. The interference pattern of our RLs has been controlled by accurately engineering the geometric properties of the photonic structures and by investigating the effects of disorder on power extraction and spectral emission. Highly collimated vertical emission was achieved in the resonant-feedback random lasing regime at ~3 THz with a maximum bandwidth of ≈430 GHz, device operation up to a 115 K heat-sink temperature, a peak optical power of ≈21 mW in pulsed mode, and up to ≈1.7 mW of CW emission. In addition to the ability to tune the laser emission frequency coarsely by varying the surface pattern distribution, we also demonstrated a new route for fine control of the spectral properties of our RLs by placing a movable mirror over the top surface in an external coupled-cavity configuration. A complex spectral dynamic was unveiled, with multiple modes simultaneously being continuously tuned over an 11 GHz range of frequencies that was further increased to ≈20 GHz with mode hopping.

## Results

### Device architecture

Our RL architecture exploits a QCL active medium sandwiched between two metallic cladding layers to create a double-metal waveguide. This confines the THz radiation tightly in the growth direction (*z*-axis) with a confinement factor nearly unitary, while light propagates in the orthogonal *x*–*y* plane, as for an almost-ideal 2D photonic resonator. To implement the random photonic structure, circular holes of radius *r* were lithographically defined on the top metal layer and vertically etched through the metal and into the highly doped semiconductor cladding (see Materials and methods); this enabled control of the optical feedback and extraction mechanisms simultaneously. THz photons undergo multiple elastic scattering events and are confined and amplified inside the active material due to the high refractive-index contrast between the gold-coated semiconductor and the air holes; furthermore, when the photon in-plane momentum is reduced to 0, light is extracted vertically through the holes and coupled into free space.

**Fig. 1:**
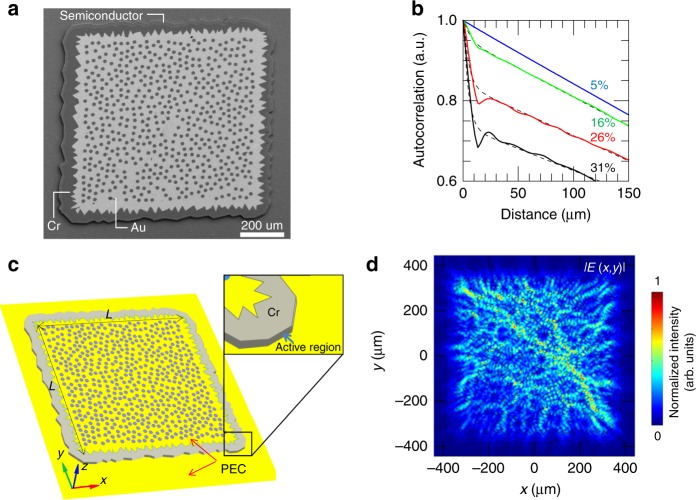
Device schematics and electromagnetic simulations. **a** Scanning electron microscope (SEM) image of a prototype RL resonator having *r*/*a* *=* 25% (filling fraction 20%) and an area of 0.57 mm^2^; the dry-etched holes penetrate both the top metal surface and the underlying highly doped contact layer. **b** Averaged spatial autocorrelation functions for random photonic structures with area of 0.57 mm^2^, for hole radii of *r* = 3 µm (*r*/*a* = 5%, filling fraction 2%) and *r* = 8 µm (*r*/*a* *=* 16%, 26%, 31% and filling fractions 8%, 21%, 30%, respectively). The autocorrelation profiles are calculated for different values of *r*/*a* to compare the characteristic ranges of disorder. The dashed lines represent the fitting function $f\left( x \right) = A\,exp\left( { - x/R_c} \right) + Bx/R_2 + C$; the sharp decay over small distances (*x* < 20 µm) is characterized by a correlation length *R*_*c*_ ≈ 3–5 μm, which decreases for larger *r*/*a* and is a signature of a strong short-range disorder. **c** Device schematic: the radius *r* and average inter-scatterer distance *a* determine the geometrical filling factor *r*/*a*, which influences both the multiple photon scattering in the resonator and the outcoupling of light to free space. The double-metal waveguide has a gold layer on the top and the bottom. A thin absorbing layer of chromium lies on the resonator border, featuring irregular protrusions with a typical scale of 20–30 µm, which is comparable with the lasing wavelength in the material (approximately 35 µm for 3 THz radiation). The QCL heterostructure was ascribed a refractive index *n*_1_ = 3.60, while a complex refractive index *n*_2_ = 4.43 + i0.31 was used for the partially lossy Cr-covered border. The Au double-metal waveguide was modelled using a perfect electric conductor (PEC) condition above and below the semiconductor mesa. The external air had *n*_Air_ = 1, and the outcoupling of light to free space was mimicked with scattering boundary conditions around the simulated region. The average size *L* of the gold patterned surface is marked on the graph. **d** Electric field modulus |**E**(*x, y*)| across the centre for the RL resonator, calculated at the half height of the mesa (*z* = 5 µm), corresponding to the computed electromagnetic mode resonating at 3.09 THz with *Q*_3D_ = 81

A Matlab code was devised to generate the hole patterns, positioning the holes in squares of side *L* = 620 µm (‘type A’ patterns) and *L* = 670 µm (‘type B’ patterns) using a uniform probability distribution given by a pseudorandom number generator. The generation algorithm implements the constraint of a minimum edge-to-edge distance of 2 µm between each pair of holes to avoid overlap between the scatterers. An increasing number, *N*, of holes in a patterned surface of average side *L* leads to a correspondingly smaller average inter-site distance *a* $= L/\sqrt N$. The geometrical filling factor of our photonic patterns was defined as the ratio *r*/*a* in analogy with that of photonic quasi-crystal lasers^18,19^. We varied *r*/*a* in the range 5–34% to explore both the effect of different degrees of scattering on light propagation in the RLs and also the outcoupling to free space, resulting from the different optical confinement of the main modes^17–19^. The corresponding filling fraction of our random resonators, determined by the area of the holes with respect to the gold-patterned resonator surface, was correspondingly varied in the range 2–36%. For *r*/*a* > 14%, a radius *r* = 8 µm was chosen to provide sufficient light scattering and extraction. By contrast, for *r*/*a* ≤ 14%, the radius was set to *r* = 3 µm to reduce the filling fraction while maintaining the inter-site distance always smaller than 100 µm so that there is a sufficient number of holes (*N* > 100) from which the light can scatter. The disordered photonic structure was then surrounded by an irregularly shaped chromium layer deposited on the mesa border featuring protrusions of typical size ≈25 µm (see Fig. 1a). The Cr layer was engineered by drawing the protrusion vertices comprised within fixed innermost and outmost limits to avoid overlap with the internal holes and so that its average width is kept comparable with the wavelengths of the expected lasing modes in the semiconductor (≈35 µm for 3 THz radiation). For resonators having different filling fractions but the same area, the Cr border was kept identical. The purpose of this partially absorbing border is to suppress undesired electromagnetic modes, such as whispering-gallery modes that have little overlap with the central random geometry; unlike the geometry proposed in ref. ^42^, here such modes are inherently suppressed by design. To ensure a good balance between surface-related diffraction and electric power dissipation, two sets of devices were designed with overall device areas (including the chromium-absorbing boundary) of 0.57 mm^2^ (type A) and 0.70 mm^2^ (type B). Figure 1a shows a scanning-electron-microscope image of a representative device.

A preliminary statistical analysis of the generated geometries can be performed by calculating their spatial autocorrelation^36^, considering a simplified two-dimensional (2D) model of the dielectric function *ε*(***r*** = *x, y*) across the mesa. For the metal-covered semiconductor region, *ε* was set equal to 1 and to 0 for the etched holes, reproducing the random spatial fluctuations of the dielectric function. The resulting autocorrelation *K* = 〈*ε*(***r***)·*ε*(***Δr***)〉 was then calculated over an ensemble of 100 different random configurations by keeping fixed the hole radius and geometrical filling factors (*r*/*a*). The autocorrelation has a typical decay length (correlation radius *R*_*c*_) that can be evaluated through fitting with an exponential function (black thin lines in Fig. 1b). As shown in Fig. 1b for type-B geometries with hole radii *r* = 3 µm and *r* = 8 µm, an increasing *r*/*a* rapidly induces a stronger short-range order, reflected in a sharper decay of the autocorrelation function. For the patterns with *r*/*a* = 5% and *r* = 3 µm, the correlation radius is approximately *R*_*c*_ ≈ 33 µm, i.e. *R*_*c*_ ≈ 0.6*a*, indicating a weak short-range disorder. For patterns with *r* = 8 µm and *r*/*a* = 16%, 26%, and 31%, however, the correlation radius reduces as *r*/*a* increases down to *R*_*c*_ ≈ 3–5 µm, i.e. *R*_*c*_ ≈ 0.1*a*–0.2*a*. The latter structures thus exhibit a far more pronounced signature of short-range disorder associated with the increased density of scatterers.

Figure 1c shows a three-dimensional schematic of an RL with a 10 µm-thick active region and a resonator area of 0.57 mm^2^, which was modelled using a commercial finite-element method (FEM) to solve Maxwell’s eigenvalue problem (see Materials and methods). The resonator supports a large number of eigenmodes with a relatively low three-dimensional quality factor *Q*_3d_ < 120 over the frequency range investigated (2.6–3.6 THz). This indicates that the generated random patterns do not provide highly selective feedback on only a few modes, but instead the disordered structure inherently acts over a broad range of frequencies. For small *r*/*a* = 5%, the numerical simulations demonstrate that the propagation of the optical modes in the resonator is characterized by a quasi-ballistic regime, as intuitively expected owing to the low hole density and the large *average* inter-site separation. Conversely, a higher *r*/*a* provides a stronger light-scattering mechanism with a much richer interference, inducing resonating eigenmodes with a complex electric field distribution; this is illustrated in Fig. 1d for a type-A device having *r* = 8 µm, *r*/*a* = 20%, and filling fraction 13%. Although both weakly localized and extended modes show a very irregular electric-field intensity profile, typically the weakly localized modes possess a larger *Q*_3d_ owing to the tighter light confinement. As there is a large number of orthogonal electromagnetic eigenmodes that partially overlap inside the resonator, local gain-mode competition plays a crucial role in the build-up of the effective lasing modes, as is typical of RLs^37^. Since it is extremely challenging to include such a quantum treatment in the FEM simulations of our RLs^45,46^ even in 2D, it is not feasible to investigate the local gain mode competition dynamics more deeply numerically, or how this influences the steady-state resonating electric fields and the resulting emission frequencies.

From the 3D simulations, we can however provide an estimate of the photon loss rate *γ*_*r*_ due to surface emission. The vertical radiative losses have been indeed included in the quality factor computation (*Q*_vertical_), to provide quantitative information on the extraction efficiency. We assume that the radiative outcoupling efficiency *η*_*r*_ is proportional to the ratio *Q*_3d_/*Q*_vertical_ being *Q*_3d_ = (1/*Q*_ohmic_ + 1/*Q*_vertical_)^−1^. From the 3D simulations we extracted an average quality factor in the operating frequency range of our RLs, *Q*_3d_ = 100, corresponding to an average total photon loss rate *γ*_3d_ ∼ 31 GHz corresponding to total losses *α*_3d_ = 3.7 cm^−1^. The computed photon loss rate due to surface emission of the main optically active modes is *γ*_r_ ~ 5 GHz, corresponding to a *Q*_vertical_ ~ 642 and consequently to an extraction efficiency *η*_r_ ~ 16%. Furthermore, the related *Q*_ohmic_ ~ 119, and then to *γ*_ohmic_ ~ 27 GHz, meaning that the main resonator photon-loss channel is expected to be the non-radiative in-plane one, (*α*_ohmic_ ~ 3 cm^−1^).

## Discussion

### Experimental data

Based on the simulation analysis, a set of QCL RLs was fabricated using a combination of optical, electron beam lithography, metal-deposition, and dry-etching techniques (see Materials and methods section); we spanned a large range of geometrical filling factors, moving from very low-density random photonic structures to closely packed configurations of scatterers. A first set of devices (type-A, area = 0.57 mm^2^) included two resonators with a fixed hole radius *r* = 3 µm, and *r*/*a* = 5% and 12% and four resonators with *r* = 8 µm, and *r*/*a* = 18%, 25%, 30%, and 33%. A second batch of lasers (type-B, area = 0.70 mm^2^) featured two resonators with hole radius *r* = 3 µm, and *r*/*a* = 5% and 12% and four resonators with *r* = 8 µm, and *r*/*a* = 20%, 26%, 30%, and 34%. A 10-µm-thick THz QCL, exploiting a hybrid, bound-to-continuum architecture with a single-quantum-well phonon extraction stage and a 700 GHz bandwidth centred at 3.05 THz was chosen as the active medium^47^.

**Fig. 2:**
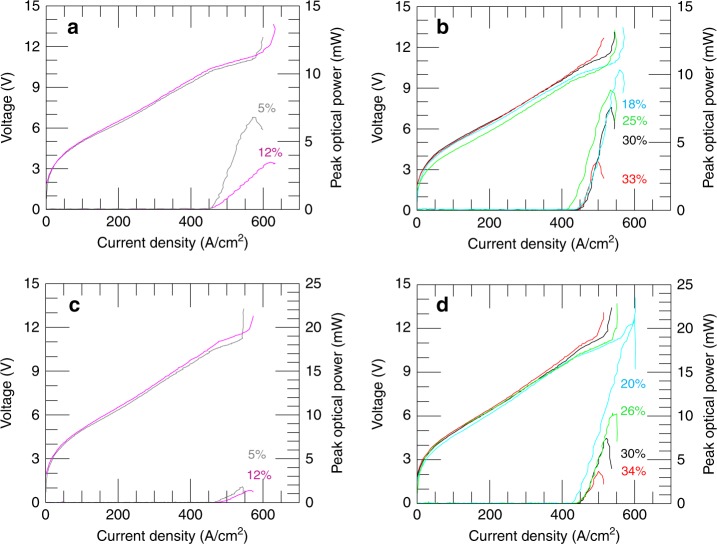
Transport characterization. **a** Plot of the current density–voltage (*J*–*V*) and current density–peak optical power (*J*–*L*) curves for RLs having an area of 0.57 mm^2^ (type A) and hole radius *r* = 3 µm. The related *r*/*a* values are labelled on the graph; the corresponding filling fractions are 2% (top curve) and 5% (bottom curve). **b** *J*–*V* and *J*–*L* characteristics for type A devices with *r* = 8 µm. The related *r*/*a* values are labelled on the graph; the corresponding filling fractions are (from top to bottom): 10%, 20%, 28%, 34%. **c**, **d** *J*–*V* and *J*–*L* curves for RLs having an area of 0.70 mm^2^ (type B) and hole radii: **c** *r* = 3 µm and **d** *r* = 8 µm. The related *r*/*a* values are labelled on the graph; the corresponding filling fractions are **c** 2% (top curve) and 5% (bottom curve). **d** (from top to bottom): 13%, 21%, 28%, 36%. All LJV measurements were acquired at a heat-sink temperature of 15 K while driving the lasers in pulsed mode with a pulse width of 200 ns and a repetition rate of 50 kHz (i.e. a 1%-duty cycle) in a nitrogen-purged environment. Optical power scales were corrected to account for the detector collection efficiency (integrating the measured optical power over the corresponding three-dimensional far-field intensity pattern) and the absorption of the polyethylene cryostat window (75%)

A comparison between the pulsed-mode current density–voltage (*J*–*V*) and power–current density (*L*–*J*) characteristics of type-A devices with hole radii *r* = 3 µm (Fig. 2a) and *r* = 8 µm (Fig. 2b) showed that for a fixed hole radius, *r*, the peak optical power decreases by increasing *r*/*a*. A maximum peak power of 7 mW was measured for the device with *r*/*a* = 5% and *r* = 3 µm and ≈11 mW for the device with *r*/*a* = 18% and *r* = 8 µm. For the latter, the differential quantum efficiency reached ≈1.5 with a maximum wall-plug efficiency (WPE) of ≈0.03%. The same comparison performed with type-B devices with *r* = 3 µm (Fig. 2c) and *r* = 8 µm (Fig. 2d), measured under the same experimental conditions of Fig. 2a, b, confirmed that the detected optical power decreases with increasing *r*/*a* at a fixed hole radius *r* as a consequences of the stronger optical confinement achieved for a larger gold-coated fraction of the top metal, with a maximum ≈21 mW for *r*/*a* = 20% and *r* = 8 µm; this laser also demonstrated the best differential quantum efficiency of 1.5 with a WPE of 0.04%.

It is worth mentioning that in the type-A RLs, the dynamic range strongly depends on the values of the geometrical filling factor (filling fraction) and the hole radius. Although the threshold current density *J*_th_ varied only slightly (*J*_th_ = 410–450 A/cm^2^) in type-A RLs for different values of *r*/*a*, which indicates the optical losses are not deeply altered within the spanned geometric parameters, the current density at the peak optical power (*J*_max_) was more strongly affected by *r*/*a* due to the sharp decrease of the optical power and overall efficiency at increasing *r/a*. With *r* = 3 µm, *J*_max_ was reached at a value of 620 A/cm^2^ for *r*/*a* = 12%, while for devices with *r* = 8 µm, *J*_max_ decreased with increasing *r/a* down to ≈520 A/cm^2^ for *r*/*a* = 33%. Similarly, for type-B devices, *J*_th_ varied between 410 and 450 A/cm^2^, but *J*_max_ showed a sharp decrease from ≈590 A/cm^2^ (*r*/*a* = 20%) to ≈520 A/cm^2^ (*r*/*a* = 34%).

**Fig. 3:**
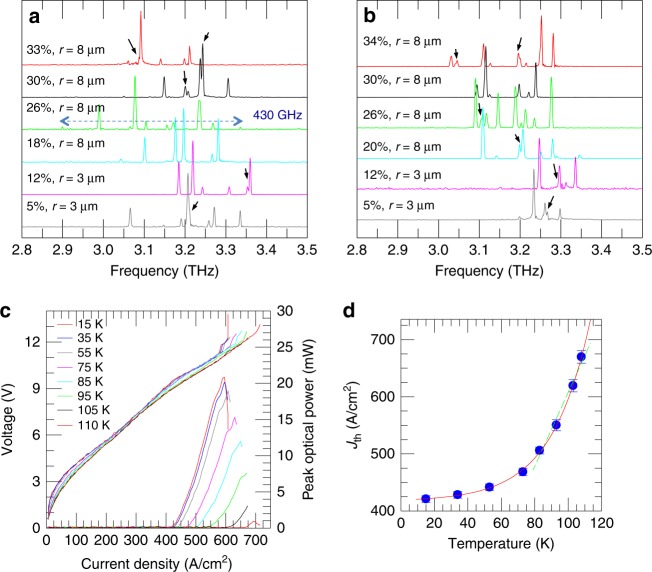
Optical characterization. **a** FTIR emission spectra of the RLs of Fig. 2a–c having different *r*/*a* (and, correspondingly, different filling fractions) and area 0.57 mm^2^ (type A). The spectra were measured in rapid scan mode using an internal DTGS pyroelectric detector. The lasers were driven with 200-ns-long pulses at a repetition rate of 50 kHz at the current corresponding to the peak power condition; the heat-sink temperature was 8 K. **b** FTIR emission spectra of the RLs of Fig. 2b–d with area 0.70 mm^2^ (type B) and different *r*/*a* (and, correspondingly, different filling fractions) measured under the same experimental conditions as (**a**). The black arrows in panels (**a**, **b**) indicate a spectral splitting of spatially overlapping modes. **c** *J*–*V* and *J*–*L* curves of the type-B laser with *r*/*a* = 20%, filling fraction 13%, *r* = 8 µm and area 0.70 mm^2^; devices were measured at different heat-sink temperatures while driving the device with 200-ns-long pulses at a repetition rate of 15 kHz (i.e. 0.3%-duty cycle). **d** Threshold current density, *J*_th_, as a function of the heat-sink temperature. The red line shows the fit with the phenomenological formula *J*_th_(*T*) = *J*_1_ + *J*_2_*e*^*T*/*T*0^, while the green dashed line is the linear fit for *T* ≥ 80 K

The FTIR emission spectra (Fig. 3a, b) show that all RLs studied here (type A, area 0.57 mm^2^, Fig. 3a; type B, area 0.70 mm^2^, Fig. 3b) feature a rich multimode emission, mostly comprising sharp, uncorrelated spectral peaks^23^. Some spectra also feature additional closely spaced spectral lines around *J*_max_ that can barely be resolved within the FTIR spectral resolution. The emitted spectral lines cover, in the best case, a 430 GHz-wide spectral bandwidth, slightly narrower than the active region gain bandwidth. This can be ascribed to the expected gain-competition mechanism in RLs, which can couple out relatively high-Q-factor optical modes with spatial overlap, but suppress the low-Q, spatially separated, modes^48^. All spectra feature a strongly variable frequency separation between adjacent spectral lines whose spacing varies from a minimum of 10–20 GHz to a maximum of 60–90 GHz. No signatures of regularly spaced Fabry–Perot or whispering-gallery modes are observed. This is as expected owing to the action of the highly disordered, absorbing chromium border that eliminates all modes extending to the edges of the device, preserving only those concentrated in the centre of the random photonic resonator. As a representative example of the rich spectrum of type-A resonators, the RL having *r*/*a* = 26% (Fig. 3a) demonstrates ten emission spectral lines spanning a 430 GHz bandwidth, amongst which a peak centred at 3.24 THz, whose half-maximum width of ≈6.5 GHz, is larger than the 3.7 GHz FTIR resolution, indicating that a number of spectrally unresolved modes may co-exist around that frequency. Amongst type-B devices, the RL having *r*/*a* = 34% features 11 distinct spectral lines (Fig. 3b), spanning a 300 GHz bandwidth. Many RLs clearly show spectral splitting of spatially overlapping modes. The characteristic presence of multiple lasing modes with uncorrelated wavefronts determines a low degree of spatial coherence of the laser emission.

Remarkably, all fabricated RLs operate up to a heat-sink temperature of *T*_H_ = 115 K, which corresponds to lattice temperature *T*_L_ ≈ 120 K^49^ (e.g. Fig. 3c) and to a characteristic temperature *T*_0_ = (107 ± 9) K (e.g. Fig. 3d), obtained by fitting the curve in Fig. 3d with the expression $J_{\mathrm{th}}\left( T \right) = J_0{\mathrm{exp}}\left( {T/T_0} \right)$. This is comparable with the *T*_0_ value measured for a standard double-metal ridge laser fabricated from the same active region (≈118 K), confirming that the high-temperature performance of the active region is not altered by the etched random photonic structure because the multiple-scattering dynamics governing the optical feedback does not perturb the thermal performance of the QCL heterostructure. The corresponding maximum peak optical power is, however, reduced from 21 mW (at 15 K) to ≈1 mW (at 110 K) as the heat-sink temperature is increased.

**Fig. 4:**
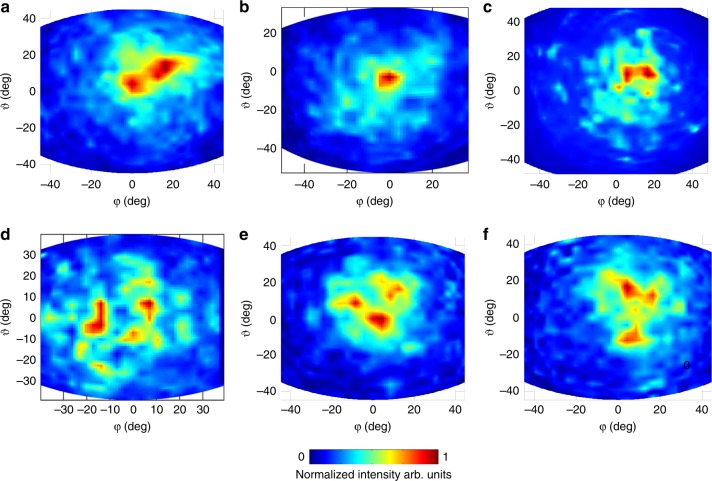
Far-field emission profiles. **a**–**c** Far-field intensity patterns measured in pulsed mode (pulse width 200 ns; repetition rate 50 kHz), at a heat-sink temperature of 15 K for RLs with area 0.57 mm^2^ (type A): **a** *r* = 3 µm, *r*/*a* = 12%, **b** *r* = 8 µm, *r*/*a* = 18%, and, **c** *r* = 8 µm, *r*/*a* = 27%, obtained by moving a pyroelectric detector on a sphere with a 5 cm radius centred on the laser surface. The corresponding filling fractions are listed in the caption of Fig. 2a–c. **d**–**f** Far-field intensity patterns measured under the same experimental conditions for RLs with area 0.70 mm^2^ (type B), **d** *r* = 8 µm, *r*/*a* *=* 20%, **e** *r* = 8 µm, *r*/*a* = 30%, and **f** *r* = 8 µm, *r*/*a* *=* 34%. The corresponding filling fractions are listed in the caption of Fig. 2b–d

The far-field intensity patterns of representative type-A RLs (Fig. 4a–c) show a well-concentrated laser emission spot covering an angular size of *Δφ* ≈ 15° and *Δϑ* ≈ 10° (*r*/*a* = 20% and *r* = 8 µm) and an almost-rectangular intensity spot with *Δφ* ≈ 20° and *Δϑ* ≈ 10° when *r*/*a* is increased up to 26%. By contrast, type-B RLs having a larger area (0.70 mm^2^), (Fig. 4d–f), have a more irregular emission with a central spot concentrated within a 10° angle (*r*/*a* = 30%, *r* = 8 µm) surrounded by two less-intense lobes. These different behaviours are determined by the complex interference scheme of the optical modes in RLs exploiting different random patterns: localized and extended modes may coexist and therefore produce an overall far-field intensity pattern that is strongly dependent on the specific filling fraction, hole radius *r* and resonator size.

**Fig. 5:**
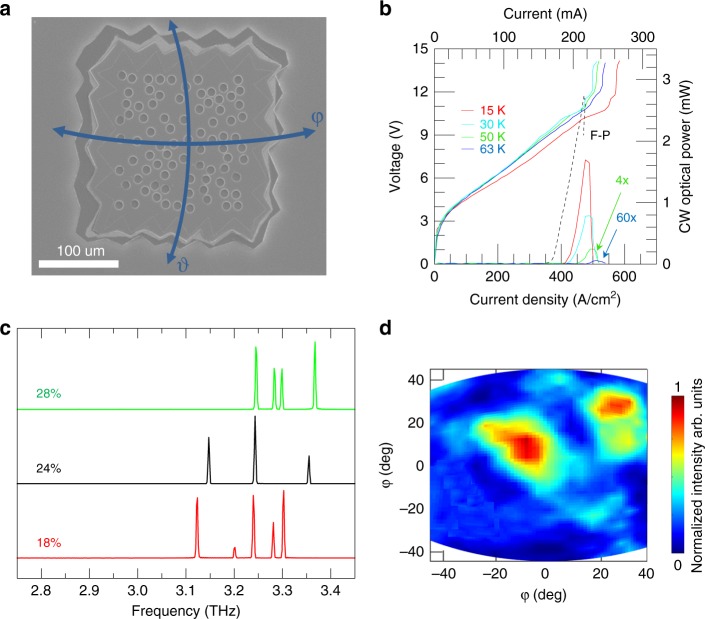
Continuous-wave emission. **a** SEM image of an RL with area 0.06 mm^2^ (type C), *r*/*a* = 28%, filling fraction 25% and *r* = 5 µm. **b** *J*–*V* and *J*–*L* curves for a type-C RL with *r*/*a* = 18% (filling fraction 10%) operating CW at heat-sink temperatures between 15 and 63 K and measured in a nitrogen-purged environment. The optical power is rescaled to take into account the absorption of the poly-4-methylpentene-1 (TPX) window (78%). The dashed line represents the *J*–*L* characteristic of a Fabry–Perot double-metal QCL having comparable area. **c** CW FTIR emission spectra for RLs having different *r*/*a*, labelled on the graph, (corresponding filling fractions 10%, 14%, 21%) and the same area (0.06 mm^2^), measured at a current corresponding to the peak output power; data were acquired with a spectral resolution of 0.125 cm^−1^, and at a heat-sink temperature of 15 K. **d** Far-field pattern emitted by a type-C RL with *r*/*a* *=* 18%, measured under the same operating conditions as (**c**), while scanning the pyroelectric detector on a sphere of 5 cm diameter. The spherical coordinates (*φ*,*θ*) employed for the far-field emission experiments are marked in panel (**a**)

To demonstrate random lasing in the CW regime, we fabricated another set of resonators (type-C) with *r* = 5 µm, different filling fractions, and a reduced overall device area of 0.06 mm^2^, as shown in the SEM image of Fig. 5a for a prototypical RL with *L* = 150 μm, *r*/*a* = 28%, and filling fraction = 25%. Although the perimeters of the mesa and the absorbing chromium border were reduced, their irregular profiles were maintained to suppress electromagnetic fields extending to the mesa edge. Indeed, the typical protrusion size of the Cr border was kept approximately 25 µm as well as its average width, while its perimeter was reduced to better fit with the smaller area covered by the photonic pattern. CW operation (Fig. 5b) was achieved with a maximum optical power of ≈1.7 mW at a heat sink temperature of 15 K, with differential quantum efficiency of 3.0 and a maximum operating temperature (*T*_H_) = 63 K, corresponding to a lattice temperature of ≈202 K^49^. For comparison, the L–I CW characteristic measured at 15 K in a Fabry–Perot (F–P) double-metal THz QCL having comparable area and fabricated from the same active region is shown on the graph. The threshold current density of the RL was 15% higher than that of the corresponding F–P laser as an effect of the increased radiative losses. Considering that the waveguide losses of double-metal waveguides are estimated to be as high as 20 cm^−1^ with typical mirror losses of the order of 1 cm^−1^, which means total losses *α*_tot_ = 21 cm^−1^, while the total losses of our RLs are *α*_tot_ = *α*_3d_ + *α*_w_ = 23.7 cm^−1^, the threshold current–density discrepancy can be attributed to the differences in the larger radiative losses produced by the extraction hole patterns implemented in the top metal. Consistently, the differential quantum efficiency of the F–P laser (2.07) is, conversely, lower than that of the RL.

The FTIR CW emission spectra (Fig. 5c) show that multimode emission covering a 200 GHz bandwidth is present in all fabricated type-C RLs, which have *r*/*a* between 18% and 28%. Similar to type-A and type-B devices, these type-C RLs also feature irregular frequency spacing between adjacent emission lines that cannot be associated with whispering-gallery or Fabry–Perot modes. The CW far-field emission profile shows multi-lobe emission with the main spot having a ≈20° divergence resulting from the different modes resonating in the miniaturized device cavity.

**Fig. 6:**
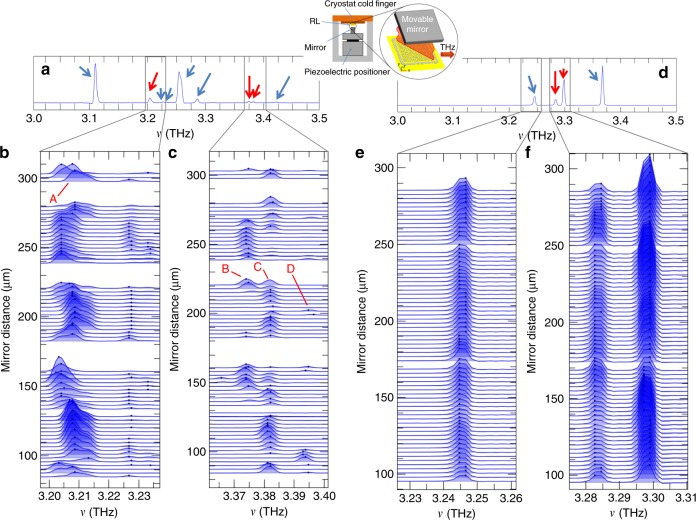
Frequency tuning. **a**–**c** FTIR emission spectra of a type-B RL with *r*/*a* = 20% (*r* = 8 µm area 0.70 mm^2^, filling fraction 13%), collected while sweeping the distance *d* of a gold mirror positioned on the top resonator surface between 80 and 310 µm and while driving the RL with *J* = 0.52 A/cm^2^ at a heat-sink temperature of 15 K with a resolution of 0.125 cm^−1^. The spectra were acquired by collecting the light guided by the external cavity (constituted by the RL surface and the parallel gold mirror) in the direction orthogonal to the RL surface, following the experimental scheme of ref. ^46^. The lasing modes are brought in and out of resonance with the bare-cavity frequencies *ν*_cav_ that are tuned according to the relation $\nu _{cav} = mc/2d$, where *c* is the speed of light in vacuum and *m* is the order of the resonance. The spectral dynamics of the lasing modes for a mirror distance in the range 80–310 µm are highlighted by showing **b** the continuous tuning of ≈11 GHz of the mode at 3.21 THz, and **c** the mode-hopping tuning of ≈20 GHz around 3.38 THz and the associated intensity quenching. All spectra are offset so that their baselines correspond to the mirror distance at which they were acquired. All spectra shown in the insets have been normalized; the blue dot indicates the position of the spectral peak. **d**–**f** CW emission spectra of the type-C RLs with *r*/*a* = 28% (*r* = 5 µm, area = 0.06 mm^2^, filling fraction 25%) collected at 15 K while varying the top mirror distance *d* between 90 and 290 µm and while driving the RL at 0.24 A. The 3.25 and 3.29 THz peaks are highlighted in (**e**, **f**), respectively, while varying the mirror distance. The red arrows identify the peaks studied in the panel below. Inset: schematics of the experimental setup. RL indicates the mounted random laser resonator

To obtain frequency tuning in our RLs, we coupled the resonators to an external gold-coated mirror positioned parallel to the top metal surface of the device and moved with a piezo driver to scan a range *d* = 80–310 µm in the *z*-direction (where 0 µm is the top of the RL), as schematically shown in the inset of Fig. 6. Laser light emitted at wavelength *λ* is expected to be reflected back by the external mirror, inducing frequency and intensity modulation of the spectral peaks when the mirror distance approaches *λ*/2, *λ*, 3*λ*/2, 2*λ*, etc.^50,51^. In an intrinsically multimode, surface-emitting RL, an even more complex spectral dynamic is expected. Indeed, coupled-mode theory indicates that each mode of the RL may exchange energy via a cross-coupling interaction with the electromagnetic modes of the external cavity. Furthermore, the different modes in the random resonator may interact as a result of mirror-induced self-coupling, especially if their frequencies are close^42^. This latter mechanism, expected in RLs, is absent in single-mode DFB QCLs^50–52^.

Figure 6a shows an example of FTIR emission spectra from a type-B RL, with Fig. 6b, c showing the evolution of the emission peaks acquired at different mirror distances in the range 80–310 µm from the top RL surface. The spectra were acquired by collecting the light guided by the external cavity in the direction orthogonal to the RL surface, following the experimental scheme of ref. ^51^. The two strongest peaks at 3.11 and 3.26 THz do not show a detactable frequency tuning within the resolution limit of the FTIR, indicating that their respective coupling strengths with the cavity are low. Instead, the less intense peaks centred at 3.21 THz (mode A) and the three peaks around 3.38 THz (modes B, C, D) undergo intensity and frequency modulation at different mirror positions. When the mirror distance approaches a resonant condition between the external cavity and the laser mode A (*d* = 94 µm, i.e. the free-space wavelength of the spectral line), a maximum continuous frequency shift of ≈11 GHz and a maximum −10 dB intensity reduction is visible in the measured spectral line. For the emission peaks B, C and D, mode-hopping is observed, with ≈20 GHz discontinuous tuning around a central frequency of 3.38 THz. By moving the mirror, a number of peaks disappear periodically while other peaks show an intensity increase in a complex interplay that depends on the matching condition for the cavity resonance as well as on the competition between modes localized inside the resonator. We performed a statistical analysis of the intensity fluctuations of the peaks, computing the intensity pair correlation *C*_ij_ between each pair of spectral lines at frequencies *ν*_i_ and *ν*_j_ in all spectra. When the correlation coefficient between two modes is positive, their intensities simultaneously grow or decrease as the mirror moves, while a negative correlation coefficient indicates that the peak intensities alternately increase or decrease, so they are competing against each other. The B and C modes consistently show a negative correlation coefficient *C*_BC_ ≈ −0.34, C and D are positively correlated (*C*_CD_ ≈ 0.36), while mode A has a negative correlation of ≈−0.47 with the most intense peak at 3.26 THz. For larger frequency spacing between the peaks, the absolute values of the intensity correlations are negligible (e.g. for A and C, *C*_AC_ ≈ −0.08), indicating that the associated intensity fluctuations are not cross-related.

The frequency tunability (11 GHz) of the spectral peak at 3.21 THz is in good agreement with the results of the fully 3D FEM simulation of the structure with transparent boundaries. Indeed, the predicted frequency-tuning excursion for a single spectral peak with an external coupled cavity is twice the photon exchange rates of the involved optical mode, as computed with no external mirror^53^. The numerical results for the computed eigenmodes indicate a photon exchange rate of the resonating mode of 5 GHz at 3.21 THz with an associated tuning excursion of 10 GHz in the presence of a movable mirror.

The other spectral peaks at 3.11, 3.24, 3.26, and 3.28 THz conversely are expected to have an associated photon exchange rate of 2 GHz, meaning that a maximum tuning excursion (4 GHz) comparable with the resolution limits of the FTIR is expected in the presence of a movable mirror.

The frequency-tunable modes additionally show a significant intensity modulation (IM). For example, spectral lines B, C, D that show a 20 GHz mode-hopping tuning range have a periodic intensity maximum modulation of −28 dB. By contrast, the two most intense emission peaks at 3.11 and 3.26 THz show a quite small −6 dB intensity modulation, consistent with the absence of frequency tuning (at a level that can be measured with the FTIR resolution available). Indeed, a poor coupling between a lasing mode and the external cavity is reflected in a small associated frequency shift and intensity modulation due to the small energy exchange between them, which means that the lasing mode is almost unperturbed by the presence of the external mirror.

Finally, we tuned the CW emission of type-C resonators. By spanning the mirror position over the range 90–290 µm (Fig. 6d–f), 4 GHz tunability of the spectral lines at 3.24 and 3.28 THz was measured, with a simultaneous maximum intensity modulation of ≈−5 dB. Similarly to the type-B RLs, the most intense spectral lines did not show detactable frequency shifts and intensity modulation, a signature of the weak coupling between them and the external cavity modes.

In conclusion, we have demonstrated broadband (430 GHz bandwidth), continuous-wave, THz RLs showing high-power (21 mW in pulsed mode, 1.7 mW in CW) directional (down to 10° divergence) beam profiles and frequency tuning, operating up to 115 K in pulsed mode and 63 K in CW. By implementing a set of non-deterministic patterns of air holes with different degrees of disorder on the QCL top metal surface, we investigated the effects induced by multiple scattering on light propagation and surface extraction. The rich multimode emission was exploited to demonstrate frequency tuning and intensity modulation of multiple modes by coupling the RL radiation to an external micro-cavity with a movable end-mirror. A maximum continuous frequency tuning of 11 GHz and discontinuous tuning of 20 GHz were achieved for different spectral peaks, as well as a periodic intensity quenching of selected spectral lines up to −28 dB.

The reported external-cavity frequency tuning and intensity modulation of multiple spectral lines make this architecture a promising platform for the development of compact multimode THz sources operating in both pulsed and CW regimes with emission spectra tunable by design. This could have applications in multi-wavelength spectroscopic sensing across the far-infrared spectral range. Together with the rich physics involved, RLs feature laser emission with an easily tunable spatial coherence^54^, which describes the correlation between waves at different points in the space, and a very high degree of second-order coherence, which is a measure of the fluctuations of the intensity. The temporal distribution of photons under diffusive scattering and no coherent feedback, above threshold, indeed exhibits Poisson statistics, like a regular laser^55,56^. In this context, electrically pumped random THz lasers can offer the highly intriguing potential to be quantum engineered to provide high radiance and spatially incoherent emission (owing to the existence of independent lasing modes with uncorrelated wave-fronts) for imaging applications free of coherent artefacts, such as spatial cross talk or speckles^57^. Furthermore, miniaturized THz RLs can be highly appealing for applications such as high-speed parallel inspection and medical diagnostics^58^ and for monitoring blood flow during treatment and surgery^59^.

As a final perspective, the proposed concept can additionally be exploited to engineer electrically driven Anderson localized THz RLs, for example, by scaling the geometry to lower-dimensional schemes, such as one-dimensional lattices that inherently tend to have much shorter localization lengths.

## Materials and methods

### Three-dimensional simulations

In our 3D model, the top and bottom metal claddings are treated as perfect electrical conductors (PECs). The 10-µm-thick GaAs-based active medium is characterized by a bulk refractive index with *n*_1_ = 3.60, both under the metal and in the regions with holes, and the external mesa border is described with a complex refractive index *n*_2_ = 4.43 + i0.31, accounting for the 7-nm-thin absorbing chromium layer placed on the top of the QCL heterostructure and providing smooth boundary conditions for the guided modes. Finally, the resonator is surrounded by air, which has an index *n*_Air_ = 1, with scattering boundary conditions being imposed on all system boundaries to mimic free-space propagation. Since the FEM model includes losses due to both the vertical extraction of light and the lateral confinement, we can simulate the overall quality factors (*Q*_3d_) associated with the electromagnetic eigenmodes.

### Fabrication procedure

The GaAs/Al_0.15_Ga_0.85_As QCL heterostructure was grown by molecular beam epitaxy on an undoped GaAs substrate. The active region features a three-quantum-well architecture, with a single extractor well^47^. The layer sequence is **5.5**/11.0/**1.8**/11.5/**3.8**/9.4/**4.2**/18.4 (in nm), where Al_0.15_Ga_0.85_As layers are shown in boldface, GaAs in roman font, and the underlined number indicates a Si-doped layer with a density of 2 × 10^16^ cm^−3^. The active region growth is terminated by a 700 nm-thick highly doped (2 × 10^16^ cm^−3^) GaAs contact layer, with an Al_0.5_Ga_0.5_As etch-stop layer on top. After growth, Au-Au thermo-compressive wafer bonding of the QCL wafer onto an *n*^+^-GaAs carrier wafer was performed. After removing the host GaAs substrate and the Al_0.5_Ga_0.5_As etch-stop layer by selective wet etching, the active region was coated with a top metal layer of Cr/Au (5 nm/150 nm). Using a combination of electron beam lithography (EBL) and optical lithography, the sample surface was patterned with air holes whose centre positions were placed with a uniform distribution in a square, which was defined using a MATLAB script. Three sets of devices were fabricated with different areas (0.57 mm^2^ for type A; 0.70 mm^2^ for type B; 0.06 mm^2^ for type C), varying the radius *r* and number of scatterers *N* to give different values of *r*/*a* in the range ≈5–34% and corresponding filling fractions in the range 2–36%. The holes are distributed in a central square region having hand-drawn protrusions having an average size of ≈25 µm and surrounded by a 30 µm-wide gold strip. To reduce cavity losses, the 700-nm-thick *n*^+^ top contact layer was totally removed from below the etched holes by means of a reactive-ion etching (RIE) process. Strongly absorbing boundary conditions were then realized by adding an external 7-nm-thin Cr border imprinted on the active region using optical lithography, with irregular protrusions of average size ≈25 µm and an average width of ≈25 µm, specifically designed to have size comparable to the lasing wavelengths in the material (≈35 µm at 3 THz). Since the Cr border acts as a protective mask for the underlying active core during the RIE process (and will be partially removed by the process), the *n*^+^ top contact layer was not etched away to ensure the suppression of modes extending towards the edge of the devices. Finally, the mesa was etched down using a second RIE process devised to optimize the vertical sidewalls of the border spikes and to avoid lateral current spreading.

After processing, individual devices were indium soldered onto a copper bar and wire bonded regularly along the perimeter to ensure uniform current injection and minimize the perturbation of the far-field emission profile.

### Optical characterization

For electrical and optical testing, the RLs were mounted on the cold finger of a liquid-helium-flow cryostat to reach the desired operating heat-sink temperatures. The data shown in the manuscript have been corrected to take into account the 75%-absorption of the cryostat polyethylene window and the collection efficiency of the detector, retrieved by integrating the pyroelectric signal obtained by a spherical far-field scan. Fourier transform infrared (FTIR) emission spectra, in rapid-scan mode with a frequency resolution of 0.125 cm^−1^, were acquired by focusing the laser radiation by means of an *f*/1 parabolic mirror through the FTIR interferometer onto an internal deuterated triglycine sulfate (DTGS) pyroelectric detector. The accuracy of the positions of the individual spectral lines was further improved by applying the zero-padding method to the interferograms. This enabled the spectral resolution to be increased to 0.0625 cm^−1^. The emitted power of CWs was measured by a calibrated pyroelectric detector and by a Thomas Keating absolute THz power-meter. The far-field emission patterns of the lasers were measured with a pyroelectric detector having a sensitive area of 7 mm^2^, which was scanned on an 8-cm-radius sphere centred on the device surface. The direction corresponding to *φ* = 0° and *ϑ* = 0° coincides with normal vector to the device surface. The piezoelectric driver used in the tuning experiment was controlled with 20 V saw-tooth pulses with an average displacement of 0.18 µm/pulse at 15 K.
